# Exosomes in the Treatment of Pancreatic Cancer: A Moonshot to PDAC Treatment?

**DOI:** 10.3390/ijms23073620

**Published:** 2022-03-25

**Authors:** Stavros P. Papadakos, Nikolaos Dedes, Alexandros Pergaris, Maria Gazouli, Stamatios Theocharis

**Affiliations:** 1 First Department of Pathology, Medical School, National and Kapodistrian University of Athens, 11527 Athens, Greece; stavrospapadakos@gmail.com (S.P.P.); ndedes@med.uoa.gr (N.D.); alexperg@yahoo.com (A.P.); 2 Laboratory of Biology, Medical School, National and Kapodistrian University of Athens, 11527 Athens, Greece; mgazouli@med.uoa.gr

**Keywords:** pancreatic cancer, exosomes, biomarkers, diagnosis, prognosis, therapy

## Abstract

Pancreatic Ductal Adenocarcinoma (PDAC) constitutes a leading cause of cancer death globally. Its mortality remains unaltered despite the considerable scientific progress made in the fields of diagnostics and treatment. Exosomes comprise of small extracellular vesicles secreted by nearly all cells; their cargo contains a vast array of biomolecules, such as proteins and microRNAs. It is currently established that their role as messengers is central to a plethora of both physiologic and pathologic processes. Accumulating data have shed light on their contributions to carcinogenesis, metastasis, and immunological response. Meanwhile, the advancement of personalized targeted therapies into everyday clinical practice necessitates the development of cost-efficient treatment approaches. The role of exosomes is currently being extensively investigated towards this direction. This review aims to summarize the current pre-clinical and clinical evidence regarding the effects of exosomal applications in the timely diagnosis, prognosis, and therapeutic management of pancreatic cancer.

## 1. Introduction

### 1.1. PDAC: Epidemiology, Pathogenesis and Current Therapeutic Management

Pancreatic cancer has long been a major public health issue and statistical figures highlight the ever-growing severity of the problem. According to GLOBOCAN 2020, 495,773 new cases and 466,003 deaths were attributed to pancreatic cancer worldwide in the year 2020 [1]. Currently, pancreatic cancer is the fourth cancer-related cause of death in the United States with the outlook being rather grim, as it is projected to be elevated to the position of second or the third place with respect to cancer mortality in Europe and North America by the end of this decade [2,3]. Incidence and mortality rates are four or five times higher in regions with a high Human Development Index such as Europe or North America when compared with countries in Sub-Saharan Africa [1]. Nonetheless, disease prognosis does not differ significantly between rich and poor countries, an observation ascribed to the lack of an effective screening process, late diagnosis, and the limited success of available treatment protocols [4]. The incidence of pancreatic cancer is slightly higher in men than in women and predominantly affects people between 60 and 80 years old with the cumulative lifetime risk of developing pancreatic cancer estimated to be around 1.5% [5,6]. It should be noted that these data are primarily derived from cases of pancreatic ductal adenocarcinoma (PDAC), which is the most common histological type (>90%) and thus is the focus of this manuscript [7]. Other less common varieties are acinar cell carcinoma or cystadenocarcinoma, intraductal papillary mucinous neoplasm with an associated invasive carcinoma intraductal tubulopapillary neoplasm with associated invasive carcinoma, mixed acinar/ductal/neuroendocrine carcinoma, pancreatoblastoma serous cystadenocarcinoma, solid-pseudopapillary neoplasm, and neuroendocrine neoplasms [8].

Regardless of histology, our understanding concerning the etiology of this disease is fundamentally lacking. As is the case with many malignancies, “modus vivendi” is a major determinant of PDAC risk. Smoking, excess body weight, and diabetes mellitus are all recognized risk factors [8,9,10,11,12,13,14,15]. Recent advances in molecular biology have served to further illuminate the mechanics PDAC. While most cases are sporadic, 5–10% are related to familial and inherited genetic disorders, such as Lynch Syndrome or Familial adenomatous polyposis, which carry a higher risk for PDAC [15,16]. Delving deeper into the genetics, mutations or deletion in genes such as PRSS1, KRAS, p16, p53, and BRCA2 are linked to an increased level of risk [17]. Of particular significance is the mutational activation of the proto-oncogene KRAS, which is harbored in up to 90% of cases [18,19]. These KRAS mutations concern mainly three hotspots, which most often are substitutions at G12 (84%). There are six possible single-base missense mutations, namely, G12D, G12V, G12C, G12A, G12S, and G12R [20]. Beyond KRAS mutations, there are a considerable number of tumor suppressors that are usually altered; some examples are CDKN2A (95%), TP53 (75%), and DPC4/SMAD4 (55%) [21,22,23,24].

Undoubtedly, the pathogenesis of PDAC remains elusive. However, a more pressing matter and a source of great concern is its insidious nature and the lack of effective treatment options. At diagnosis, PDAC is usually at least 2–4 cm in diameter and has in many occasions already infiltrated the surrounding structures and lymph nodes [8]. Unfortunately, just 10–15% of patients are candidates for surgery, which is associated with a 5-year-survival rate of around 25% [24,25,26]. The chance of survival is significantly better when surgery is combined with chemotherapy [27,28,29]. While the survival rates after surgery are not impressive, they are considerably higher than those of patients who present with metastases at diagnosis, which are no more than 2.7% [29]. For those who are not candidates for surgery and who have an Eastern Cooperative Oncology Group (ECOG) Performance Status (PS) between 0 and 1, the first treatment option is chemotherapy with leucovorin, fluorouracil, irinotecan, and oxaliplatin (FOLFIRINOX) or gemcitabine plus nab-paclitaxel. Patients with a PS of 2 generally receive gemcitabine alone, while treatment for those with a PS of 3 is generally limited to the best supportive care. Further treatment options are available for a limited number of patients with NTRK fusions, BRCA mutations, or those who are mismatch repair deficient or whose disease has high microsatellite instability [30]. Nevertheless, the available therapeutic strategies have limited efficacy and the disease usually develops resistance to even the most active agents, such as gemcitabine [28,31]. Currently, the end result is disheartening: patient median survival time is less than 6 months [32]. This state of affairs calls for a different approach to PDAC diagnosis and management, and one of the novel strategies is the manipulation of exosomes [33,34,35,36,37,38]. This review provides a precis of exosome biology and its possible applications to the therapeutics of PDAC.

### 1.2. Extracellular Vesicles: Characteristics and Roles in Physiology and Disease

Extracellular vesicles (EVs) are membrane-bound lipid particles secreted by all cells and function as a means of communication between the parent and the recipient cell through their biological cargo (proteins, nucleic acids, lipids, and metabolites). With regard to their classification, the International Society of Extracellular Vesicles recommends the division of EVs based on their size: medium/large EV (>150 nm) and small EV (<150 nm) [39]. Nonetheless, there is no general consensus regarding the categorization of EVs in the current literature due to the considerable overlap of vesicle sizes. However, most researchers discern three major groups of EVs: small EVs or exosomes, microvesicles, and apoptotic bodies.

**Small EVs or Exosomes:** Exosomes are nanoparticles with a diameter measurement in the range of 30–150 nm. The first step in the biogenesis of exosomes is the inward budding of the extracellular membrane, which forms the endosome. The endosome undergoes inward invaginations and thus encloses biological cargo, which produces the intraluminal vesicles. The total number of intraluminal vesicles and the endosome in which they are contained comprise a multivesicular body (MVB). Once formed, these MVBs can either fuse with lysosomes and have their content degraded or release their content to the extracellular matrix through the endosomal sorting complex required for the transport- (ESCRT)-dependent or the ESCRT-independent pathway. Among exosomes, there are several common features such as the tetraspanin family of surface proteins (CD9, CD63 and CD81), integrins, heat shock proteins, ESCRT, actin and flotillins, while other molecules are specific to the donor cell, such as type MHC Class I and II. The end result is the activation of a variety of signaling cascades, which are determined by the interconnection between exosome surface proteins and receptors on the recipient cells [39,40].

**Microvesicles:** The sizes of these vesicles range from 50 nm to 1μm and are generated by the outward budding of the cell membrane [41]. Their content is similar to that of exosomes, and they are considered to be involved on tissue homeostasis (regeneration, angiogenesis, cell cycle regulation, inflammation, etc.) [42,43].

**Apoptotic Bodies:** These vesicles comprise large fragments of a cell which has undergone apoptosis. They are the largest subset of the EVs with a size of up to 2 μm and therefore provide a substantial molecular pool for recipient cells [44]. Apoptotic bodies are readily cleared by macrophages, which recognize phosphatidylserine on the surface of the EV in question; the defects of this process are linked to auto-immune reactions [45]. Similarly to microvesicles, apoptotic bodies play a role in tissue homeostasis, such as bone metabolism [46].

### 1.3. Exosomes

Classically, exosomes were considered as a means for the disposal of nonessential cellular cargo. The first documentation of the term “exosome” in the literature was in a publication delineating the maturation of reticulocytes [47]. The development of more advanced cell culture techniques in conjunction with the improvement in exosomal isolation capacity and immune-electron microscopy has led to a deepening in our perception of the underlying biosynthetic and regulatory mechanisms of exosomes [48]. It is now common knowledge that they are important mediators of intercellular communication under both normal and diseased conditions [49]. In more detail, the mediation of exosomes regulates a wide variety of physiologic processes: skin regeneration [50], bone remodeling [51], placental formation [52], and organ crosstalk during exercise [53]. It also serves as a diagnostic and predictive biomarker in inflammatory diseases such as rheumatoid arthritis [54], systemic lupus erythematosus [55], and nephropathies [56].

Similarly, the participation of exosomes in cancer pathophysiology with applications in diagnosis, prognosis, and treatment has been extensively studied [57,58,59].

Focusing on PDAC, exosomes have the potential to help determine treatment decisions. One such application is the perspective given by liquid biopsy, as was hinted by Buscail et al. who demonstrated that the level Glypican-1 positive exosomes circulating tumor cells and CA19-9 are able to be used for diagnostic and prognostic purposes in patients with resectable tumors [60,61]. Moreover, Nakamura et al. found that extracted exosomes from pancreatic juice could be used to discriminate PDAC from chronic pancreatitis [62]. Furthermore, treatment adjustments could be made by monitoring exosomal miRs, known to confer chemoresistance [63].

### 1.4. Artificial Exosomes: Engineering and Applications

Exosomes possess abilities and characteristics that render them a highly promising drug delivery system. However, the utilization of natural exosomes comes across a variety of technical issues such as difficulties in large-scale production, isolation, drug conjugation, stability, and quality control. To compensate for these deficiencies, a considerable effort is being made that aims to develop artificial exosomes by means of nanobiotechnology. With respect to the production of these vesicles, many different approaches have been suggested, all of which can be summarized into three distinct filter categories, namely, “top-down”, “bottom-up,” and “biohybrid” technologies [64].

The “top-down” strategy aims at the creation of nanovesicles through cell manipulation. This can be achieved with multiple techniques such as forced passage through porous membranes or microfluidic devices, nitrogen cavitation, cell membrane blebbing by virtue of sulfhydryl-blocking, and exposure to alkaline solutions followed by sonication. The major advantage of the “top-down” methodology is that the produced nanovesicles are fully biological with similar features to natural exosomes. Unfortunately, their production process also involves cell destruction, which limits its production sustainability and is associated with the presence of various contaminants [65,66,67,68].

On the other hand, “bottom-up” methodologies produce exosome-mimetics by supramolecular chemistry, which combines basic components such as lipids or proteins in a progressive manner so as to form complex structures [64]. This approach offers a pure product that is eligible for mass production, while its most substantial shortcoming is attributed to the difficulties in the liposome modification necessary in order to accomplish the sufficient mimicry of natural exosomes [69,70].

Finally, biohybrids, a form of exosome-mimetic, can be created through the merging of synthetic nanoparticles with natural vesicles, which is achievable by freeze-thawing, incubation, or co-extrusion [64]. This strategy might produce a carrier with higher efficiency than liposomes and higher stability than natural exosomes. Regrettably, this methodology is the most resource-intensive [71].

**Figure 1:**
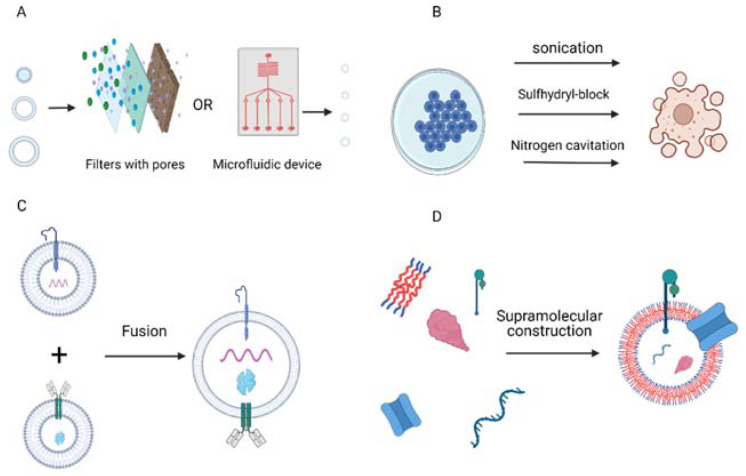
The construction of artificial exosomes (**A**,**B**), the “top-down” strategy: forced passage through porous membranes or microfluidic devices, nitrogen cavitation; cell membrane blebbing. (**C**) The supramolecular construction of artificial exosomes with biohybrid technologies (**D**), the “bottom-up” methodology: the supramolecular construction of complex structures from basic components. Created with BioRender.com.

In summary, the products of nanobiotechnology are expected to comprise a major drug delivery system in the near future and bring medicine closer to personalized therapy. However, this field is still in its early stages of evolution. Therefore, further research is needed to improve production protocols, devise characterization methods, and achieve a meaningful outcome. The above-mentioned techniques are presented in Figure 1.

## 2. PDAC Microenvironment: Role in Neoplasia

The PDAC’s microenvironment consists of a wide variety of heterogeneous cell populations, namely, endothelial cells and pericytes comprising the vascular compartment, extracellular matrix (ECM)-producing cells, neuroendocrine cells, and immune and inflammatory cells at various activation states. From a review of the preceding literature, it is apparent that their contribution to tumorigenesis has long been underappreciated. This is reflected by the utilization of nonspecific chemotherapeutic agents in PDAC’s therapy in order to achieve cell-cycle arrest. With the broadening of our understanding of PDAC’s initiation, proliferation, and metastatic potential as well as of the dynamic interconnection between tumor cells and the cells of tumor microenvironment (TME), new target-specific therapeutic approaches are under development. This complex network is interconnected with physical interactions, immune suppression, and pleiotropic crosstalk mechanisms [72]. This interconnection is mediated through exosomal intercellular communication.

As far as a tumor’s physiology is concerned, the PDAC cells are crowded in an intense stroma of fibrous tissue, which is characteristic of the disease [73]. The immune cell infiltrates constitute nearly 50% of the tumor mass [74]. A glycosaminoglycan desmoplastic reaction (analyzed below) is responsible for the notable increase in interstitial fluid pressure (IFP) [75], which constricts the vascular inflow, creating an oxygen-deprived, nutrient-poor environment [72]. Under hypoxic conditions, the pancreatic cancer cells generate and secrete exosomes carrying miR-301a-3p, which polarize the stromal macrophages into a M2 phenotype upregulating the PTEN/PI3K signaling pathway [76]. Moreover, Yang et al. demonstrated that M2 macrophages had a positive correlation with the microvessel density of human PDAC. M2 macrophage-derived exosomes were more enriched in miR-155-5p and miR-221-5p compared to M0 macrophage-derived exosomes, which promoted the angiogenic ability of endothelial cells in an E2F2-dependent manner [77]. Similarly, hypoxic PDAC cell-derived exosomal miR-30b-5p was found to promote angiogenesis through the inhibition of GJA1 in a mouse model. This observation holds the promise of becoming the basis of the development of a new diagnostic marker, as patients with PDAC had higher levels of total miR-30b-5p and exosomal miR-30b-5p in peripheral blood plasma than did healthy subjects [78]. Hypoxia is also targeted by modern therapeutics. For example, evofosfamide is reduced in hypoxic conditions, releasing its active metabolite bromo-isophosphoramide (Br-IPM). Br-IPM exerts its cytotoxic effects preferentially in the hypoxic areas of the tumor and potentiates the utilization of radiotherapy and chemotherapy as adjunct therapeutic modalities [79]. However, the TME and the disease are not static, as the transfer of miRNA in macrophage-derived exosomes induces drug resistance in PDAC [80]. Exosome-mediated intercellular communication is also central to disease progression. It has been recently found that the liver metastatic niche is prepared in advance by the PDAC-derived exosomes carrying the CD44v6/C1QBP complex, which causes hepatic fibrosis [81]. In this context, exosomal long noncoding RNA LINC01133 was demonstrated to promote the proliferation, migration, invasion, and epithelial-mesenchymal transition of pancreatic cancer cells [82].

Beyond mere description of disease pathogenesis, the exploration of exosome-mediated communication might uncover a new basis for potential treatment. Based on this thesis, Zhou et al. created an exosome-based dual delivery biosystem for enhancing PDAC immunotherapy and reversing the tumor immunosuppression of M2 macrophages through the disruption of the galectin-9/dectin 1 axis [83]. TME was also targeted in a recent clinical trial which tested the ibrutinib plus nab-paclitaxel/gemcitabine versus the placebo plus nab-paclitaxel/gemcitabine. Unfortunately, no statistically significant increase in survival was noted [84]. Alternation of the TME and immune system activation has also been attempted via the use of a modified herpes simplex virus by Wang et al., while the same aim was pursued by Jiang et al. through MEK and autophagy co-inhibition, coupled with CD40 agonism [85,86].

**Figure 2:**
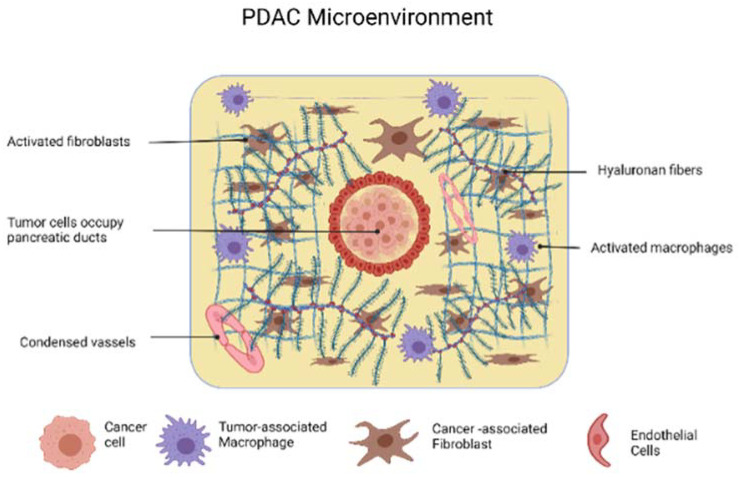
The PDAC microenvironment. Created with BioRender.com.

Taking all of this into consideration, PDAC TME is central to pathogenesis and the physical history of the disease. These processes—mediated largely by exosomes—are becoming ever more defined. Thus, the next step is to utilize the attributes of exosomes and to manipulate them in such a way that they become clinically meaningful. A brief synopsis of the above is presented in Figure 2.

## 3. Therapeutic Applications of Exosomes in PDAC

The urgency regarding the generation of a more personalized approach to medical diagnosis and treatment has placed exosome technology in the spotlight. The main applications that are under investigation concern the reduction of drug resistance, the utilization of exosomes as drug-carries, the therapeutic modification of the immune tumor microenvironment, and the targeting of KRAS. The contributions of exosomes in the therapy of PDAC will be presented below.

### 3.1. Chemoresistance in PDAC Treatment

Resistance to chemotherapy is an appreciable problem in the treatment of pancreatic cancer, and there is growing evidence that exosomes are crucially involved. The underlying mechanisms for nontargeting drugs include the upregulation of multidrug resistance proteins (MDR) which comprise several classes of drug efflux pumps such as P-glycoproteins (P-gp), multidrug-resistant protein-1 (MRP-1), and ATP-binding cassette sub-family A member 3(ABCA-3). They also include the enhancement of proliferating and anti-apoptotic pathways, the downregulation of drug targets, and the mutational alteration of drug targets. The latter also constitutes the leading cause of drug resistance in targeted therapies [87]. Exosomes contribute to drug resistance either directly (by carrying the chemotherapeutic agents outside of the cancer cells [88]) or indirectly (through their cargo-mediated effects); they derive from chemotherapy-resistant cancer cells and the cells of TME and modulate the phenotype of chemotherapy-sensitive cancer cells [87]. In recent years, preclinical data on the role of exosomes in chemotherapy resistance have increased remarkably.

Radical oxygen species (ROS) generation is a well-documented precipitant of cell death. Patel et al. documented that among the EVs, exosomes contribute the most to gemcitabine (GEM) resistance by conveying superoxide dismutase 2 (SOD2) and catalase (CAT) to detoxify ROS. In parallel, the exosomally transmitted miR-155 inhibits the expression of deoxycytidine kinase (DCK), deescalating the phosphorylation of GEM monophosphate to its active metabolite. The inhibition of mir-155 or the downregulation of DCK counteracts the exosome-induced GEM resistance [89]. Mikamori et al. shed more light on the effects of miR-155 on PDAC. They demonstrated that miR-155 enhances the secretion of exosomes, modifies their cargo, increasing its excretion, and induces GEM resistance through suppressing apoptosis by downregulating TP53INP. The interception of RAB27B’s function mitigated drug resistance by enhancing the caspase 3/7-induced apoptosis [90]. These results indicate that miR-155 in exosomes could be a potential therapeutic target. Taking a step further, survivin exerts its anti-apoptotic effects by binding and deactivating caspase-9 through the phosphorylatable Thr. Since the utilization of Survivin-T34A by adenoviral vectors showed moderate therapeutic results [91], Aspe et al. treated MIA PaCa-2 cell lines with Survivin-T34A-enriched exosomes raised by engineered melanoma cells. They reported that the combination of GEM and Survivin-T34A-enriched exosomes ameliorated nearly three times the apoptotic process compared to GEM or exosomes alone [92]. Although the role of the EPH/ephrin system on cancer has been thoroughly presented [93], we are obligated to indicate the significant role of exosomal EPHA2 in GEM resistance. A comparative analysis of the protein exosomal cargo indicated a 5-fold increase of EPHA2 in the GEM-resistant PANC-1 cell line in comparison with the more GEM-sensitive MIA PaCa-2 and BxPC-3 lines. The silencing of the EPHA2 in PANC-1 cells improved GEM resistance by approximately 25% [94]. In the complex TME, drug resistance can also be imposed by noncancerous cell populations.

**Figure 3:**
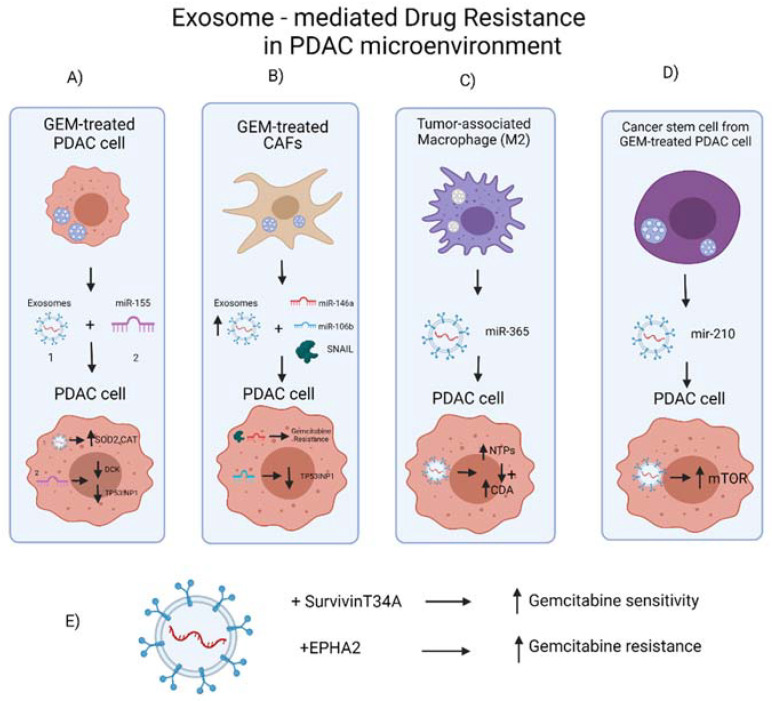
The contribution of the PDAC microenvironment to the generation of drug resistance. (**A**) GEM-treated PDAC cells secrete miR-155 through exosomes enhancing the antioxidant capacity of cancer cells (through the downregulation of SOD2 and CAT), disrupting the activation of GEM (through the downregulation of DCK) and cellular stress-sensing systems (by upregulating TP53INP1). (**B**) GEM-treated CAFs exosomally secreted miR-106b, miR-146a, and Snail, all of which mediate their intrinsic resistance to GEM. (**C**) The M2-derived exosomally excreted miR-365 increased the intracellular NTP pool and the CDA. (**D**) GEM-resistant cancer stem cells secreted through exosomes miR-210 induced drug resistance through mTOR signaling potentiation. (**E**) The knockdown of EPHA2 in drug-resistant cells decreased their efficiency in transmitting GEM resistance. Created with BioRender.com.

Excepting the metabolic support that Cancer-associated fibroblasts (CAF) provide to the cancerous epithelial cells, Richards et al. documented that CAFs, when exposed to GEM, tend to transmit their innate resistance to GEM with exosomes highly enriched in miR-146a and Snail. Blocking the secretion of exosomes with GW4869 enhances PDAC cell killing by GEM in co-cultures and shrinks the tumor volume [95]. Analogously, miR-106b-enriched exosomes of CAF origin promote resistance upon GEM exposure, combining with TP53INP. Those actions can be alleviated by miR-106b inhibitors [96]. Tumor-associated macrophages (TAMs) and cancer stem cells are also cell populations which significantly contribute to GEM resistance. M2-derived exosomes carried circa ten times more miR-365 in comparison to M1-originated exosomes, conferring a 2.6-fold increase in the intracellular NTP pool. This stimulates the upregulation of cytidine deaminase (CDA) in the PDAC cells, which in turn deactivates GEM while the elevated dCTP concentration antagonizes dFdCTP. Genetically modified Rab27 a/b knockout mice, which are unable to secrete exosomes, metabolize GEM better than the wild-type mice, while the treatment of PDAC-carrying mice with miR-365 oppressors restored sensitivity to GEM [80]. Exosomes derived from GEM-resistant cancer stem cells are abundant in miR-MiR-210 and contribute to GEM resistance, potentiating the mTOR signaling pathway [97]. All the above are summarized in Figure 3.

### 3.2. Exosomes as Drug Carriers

The rationale for using exosomes as drug carriers in the treatment of pancreatic cancer stems from the similarity of their cell membranes to those of human cells. This trait is responsible for some advantageous properties, including favorable bioavailability and biocompatibility, inconsequential immunogenicity, the enhancement of drug release stability, the expansion of a drug’s half-life, and the ability to penetrate biological membranes. Τhe end result is the development of more effective drugs with improved profiles of side effects [98].

As mentioned above, the administration of drugs to cell populations of mesenchymal origin (e.g., adipose tissue, bone marrow) can trigger the generation and secretion of exosomes. This feature is exploited in order to create a model where chemotherapeutics are incorporated in mesenchymal tissues and secreted with exosomes. Another technique utilizes modern nanotechnology to load or pulse drugs directly into exosomes. Table 1 and Table 2 summarize the preclinical data regarding the effects of certain chemotherapeutics and of miRNA mimetics, respectively.

### 3.3. Immune Microenvironment Modifications

The discovery of exosomes and the evolution of their isolation and processing technology has transformed the logic of cancer vaccination. The initial vaccine development efforts aimed at stimulating antigen-presenting dendritic cells with cancer-specific antigens in order to create vaccines that targeted distinct cancer cell populations [112]. An increasing understanding of exosomal function improved the specificity of the vaccines and enabled the co-administration of drugs while in parallel, expanding the scope of therapeutic interventions within the immune microenvironment of the tumor. This was accomplished by the enhancement of immunotherapy; the utilization of modified exosomes as vectors in photodynamic therapy and their contribution to the reprogramming of the TME. In recent years, significant preclinical data have emerged in this direction.

Wang et al. demonstrated that under hypoxic conditions, PDAC cells are urged in an HIF-1a/2a-mediated manner to upregulate the expression of miR-301a-3p. The secretion of miR-301a-3p-enriched exosomes inhibits PTEN expression and potentiates the PI3K signaling pathway to polarize macrophages towards an M2 phenotype. The end result is a statistically significant association of miR-301a-3p with a more aggressive clinicopathological disease regarding the extent of tumor invasion, metastatic potential, perineural infiltration, and TNM staging. The silencing of miR-301a-3p reverses those effects [76] and could possibly be combined with antifibrotic treatments. The phenotype switching of macrophages may have enormous therapeutic implications. Su et al. reported that exosomes derived from miR-155 and miR-125b-2-transfected Panc-1 cells could shift macrophages from an M2 towards the immune-stimulatory M1 phenotype [113]. While Que et al. documented that PDAC cell-originated exosomes, in which the miRNA cargos were lysed, could more effectively activate dendritic cells and cytokine-induced cell killer populations [114], Xiao et al. incorporated all-trans-retinoic acid, GEM, and sunitinib into pancreatic cancer-derived exosomes and into dendritic cells in a continuum. This coalescence was able to target myeloid-derived suppressive cells (MDSCs) at a multitude of developmental stages, exhibiting prolonged survival and an improved metastatic profile [115].

These scientific efforts led to a more comprehensive theranostic approach. Yang et al. modified MIA-PaCa-2 cell-derived exosomes loading Chlorin e6 (Ce6), a potent photosensitizer. Ce6 can enhance the photoacoustic signals and trigger ROS generation upon exposure to laser irradiation. Additionally, potentiating the local antigen-presenting cells releases a cytokine reaction which generates an immune reaction by CD8 T cells. This combination of image-guided photodynamic therapy and immunotherapy showed promising results in mice with minimal side effects [116] and should enter into the stage of clinical trials. On the other hand, Zhou et al. utilized exosomes derived from bone marrow mesenchymal stem cells (BM-MSC) and, via electroporation, inserted galectin-9 siRNA and oxaliplatin. The latter exerts its action directly on pancreatic cancer cells by causing apoptosis and indirectly through causing immunogenic cell death. The enhancement of the immunological response is also achieved by the silencing of galectin-9, which polarizes macrophages towards an M1 phenotype and contributes to the aggregate cytotoxic response [83].

### 3.4. Targeting KRAS in PDAC

Despite the abovementioned fact that *KRAS* mutations drive carcinogenesis and are existent from early adenomatous lesions to late metastatic disease, there were not until recently any KRAS-targeting therapies. In fact, for decades, KRAS was considered “undruggable” based on the immense affinity of Ras for the highly abundant GDP,GTP in the cell’s cytoplasm [117]. Numerous approaches to downregulate the Ras signaling pathway were engaged. These are reviewed in depth elsewhere [118], and we intend to introduce only the fundamental pharmacologic mechanisms. Since the attempt at the allosteric inhibition of KRAS encountered difficulties, research efforts have turned towards the displacement of KRAS from the plasma membrane, the inhibition of downward signaling molecules, and the percussion of the cancer cell’s metabolic repertoire [118]. Recently, Kesler et al. discovered the druggable S I/II-pocket, a lipophilic cleft which could, upon pharmacologic inhibition, arrest the GTPase-activating protein (GAP), guanine nucleotide exchange factors (GEFs), and the downstream synergies of KRAS [119], potentially offering a pan-RAS inhibitor, while the first switch II-pocket inhibitors have gone into clinical trials [120]. In parallel, significant progress has been made in the development of exosome-based therapeutic applications.

Τhe production of cutting-edge biotechnological therapeutic products often runs into the inability to produce sufficient quantities to cover patients’ needs. Mendt et al. generated siRNA-loaded exosomes to an extensive scale. The KrasG12D-silencing RNAs were incorporated via electroporation into mesenchymal-derived exosomes inhibiting the PDAC tumor growth in mouse xenografts. After intraperitoneal administration, iExosomes were allocated preferentially in pancreatic tumor tissue in comparison to the neighboring organs with a mechanism which remains unclarified [121]. The KRAS-driven enhancement of micropinocytosis [122] might be a contributing mechanism. The prolongation of survival is documented in several PDAC mouse models [121]. In the same direction, Kamerkar et al. documented the superiority of CD47-anchored fibroblast-derived exosomes when compared with liposomes. The “don’t eat me” signal conferred by CD47 enhanced the exosome’s bioavailability, significantly limiting the side-effects profile. The incorporation of KRASG12C-silencing RNAs significantly shrank the tumor volume in the mouse models of orthotopic tumors [122]. These encouraging results led to a Phase I clinical trial. Patients with Stage IV clinical disease will be subjected to the administration of KrasG12D siRNA-carrying MSC-derived exosome at days one, four, ten, and every two weeks for three courses. The results are expected to be released soon [123].

## 4. Conclusions—Future Perspectives

The use of exosomes in clinical practice has the potential to become an important weapon in our therapeutic armory, capable of serving the demands imposed by personalized precision medicine. Although the consolidation of evidence concerning the importance of exosomes is relatively recent, a significant amount of preclinical data has begun to accumulate into the literature and a modest fraction of this is directed into clinical trials in order to test its diagnostic [124] or therapeutic capacity [123]. In parallel, the generation of a PDAC organoid which accurately recreates a tumor’s ecosystem has the potential to notably enrich the scientific community with data generated from the interactions among several cell populations [125]. The exosomally delivered KrasG12D siRNA constitutes the first KRAS-targeting therapy, and if it succeeds, it could transform the architecture of PDAC’s treatment algorithms, while the favorable usage profile of exosomes with an extensive distribution in body fluids and a minor triggering of immune responses [126] could render them ideal carriers of the established chemotherapeutics paclitaxel [99,102], GEM [100,101], and repurposed natural extracts such as curcumin [103]. Scientific advancements have given us the means to engineer exosomes providing theranostics technological applications [83,116] which combine diagnostic and radicalized treatment avenues such as the modification of a tumor’s immune ecosystem in conjunction with photodynamic therapy (PDT) [116]. The continual delineating of drug-resistance mechanisms elucidates the principal role of exosomes in the process and could ideally offer new treatment-enhancing targets. Finally, given the implications exosomes hold in the generation of the pre-metastatic niche [127,128], the introduction of exosome inhibitors might revolutionize therapy for metastatic disease, which is the main cause of death in cancer patients.

## Figures and Tables

**Table 1:** The effects of chemotherapeutics utilized against PDAC in in vivo and in vitro experiments.

Drug	Origin of Exosome	Cell Line/Animal	Results	References
Paclitaxel	Mesenchymal	CFPAC-1 cells	Less than 10% greater IC50 of PTX-MV vs. PTX	[99]
GEM	Mesenchymal	CFPAC-1 cells	BM-MSCsGCB-CM has 40% less V50 than pMSCsGCB-CM	[100]
GEM	Autologous	Panc-1 cells/BALB/c nude mice	**At day 16 (after treatment):** ExoGEM 10 mg/kg-50% lower tumor volume	[101]
			**At day 16 (after treatment):** GEM 50 mg/kg-2 fold increase in tumor volume	
GEM and Paclitaxel	Bone Marrow-MSC	MiaPaca-2 cells/nude mice	**Survival:** Exo-GEMP-PTX group: 88 days Exo group: 42 days GEM group: 60 days Exo-GEM group: 63 days GEMnab-PTX group: 73 days	[102]
Curcumin	Autologous	PANC-1 and MIA PaCa-2 cell lines	**PANC-1 cells at 72 h:** curcumin-positive exosomes 50% cell killing vs. curcumin-negative exosomes 0% cell killing	[103]
			**MIA PaCa-2 at 72 h:** curcumin-positive exosomes 60% cell killing vs. curcumin-negative exosomes 0% cell killing	

**Table 2:** The preclinical data regarding the impact of certain exosomal miRNAs in PDAC.

Drug	Exosome Origin	Cell Line/Animal	Pathway/Function	Outcomes	References
miR-1231 mimics	BM-MSCs	BxPC-3 and PANC-1/female BALB/C nude mice	EGFR, Cyclin E	Downregulation	[104]
			Wound healing	Deterioration	
			Invasion	Deterioration	
			Tumor volume	Deterioration	
miR-126-3p mimics	BM-MSCs	PANC-1 cells	Proliferation, migration, invasion	Deterioration	[105]
			Apoptosis	Increase	
			ADAM9	Downregulation	
			Growth rhythm/Tumor volume	Decrease	
miR-145-5p mimics	Umbilical cord-MSCs	Capan-1, CFPAC-1, BxPC3 and Panc-1 cell lines/nude mice	SMAD3	Suppression	[106]
			Tumor proliferation	Decrease	
miR-27a inhibitors	PC cell-derived	PDAC cell lines: H6c7, SW1990, Capan-1, BxPc-3 and PANC-1 and microvascular endothelial cell line: HMEC-1/nude mice	BTG2	Upregulation	[107]
			proliferation, migration, invasion, angiogenesis	Suppression	
			Apoptosis	Increase	
GW4869	Pancreatic stellate cell	PANC-1 and Suit-2 cell lines	Proliferation, migration	Increase	[108]
			CXCL-1,-2	Increase	
RTK inhibitors	Cancer-initiating cell (CIC)	Capan-1, A818.4 cell lines	CD44v6kd non-cic, Tspan8kd non-CIC	Alterations	[109]
			Survival	Increase	
			Tumor cell invasion	Suppression	
miR-501-3p antagomiR	M2 macrophages	PANC-1, BxPC-3/male BALB/c nude mice	Tumor volume	Suppression	[110]
			Metastatic burden	Suppression	
			TGFBR3	Increase	
miR-125b-5p inhibitor	PC-1.0 derived (greatly invasive)	PC-1, PC-1.0 cells	STARD13	Downregulation	[111]
			Migration, invasion	Suppression	
